# The Yeast Rab GTPase Ypt1 Modulates Unfolded Protein Response Dynamics by Regulating the Stability of *HAC1* RNA

**DOI:** 10.1371/journal.pgen.1002862

**Published:** 2012-07-26

**Authors:** Nikoleta G. Tsvetanova, Daniel P. Riordan, Patrick O. Brown

**Affiliations:** 1 Department of Biochemistry, Stanford University School of Medicine, Stanford, California, United States of America; 2 Howard Hughes Medical Institute, Stanford University School of Medicine, Stanford, California, United States of America; University at Albany, State University of New York, United States of America

## Abstract

The unfolded protein response (UPR) is a conserved mechanism that mitigates accumulation of unfolded proteins in the ER. The yeast UPR is subject to intricate post-transcriptional regulation, involving recruitment of the RNA encoding the Hac1 transcription factor to the ER and its unconventional splicing. To investigate the mechanisms underlying regulation of the UPR, we screened the yeast proteome for proteins that specifically interact with *HAC1* RNA. Protein microarray experiments revealed that *HAC1* interacts specifically with small *ras* GTPases of the Ypt family. We characterized the interaction of *HAC1* RNA with one of these proteins, the yeast Rab1 homolog Ypt1. We found that Ypt1 protein specifically associated *in vivo* with unspliced *HAC1* RNA. This association was disrupted by conditions that impaired protein folding in the ER and induced the UPR. Also, the Ypt1-*HAC1* interaction depended on *IRE1* and *ADA5*, the two genes critical for UPR activation. Decreasing expression of the Ypt1 protein resulted in a reduced rate of *HAC1* RNA decay, leading to significantly increased levels of both unspliced and spliced *HAC1* RNA, and delayed attenuation of the UPR, when ER stress was relieved. Our findings establish that Ypt1 contributes to regulation of UPR signaling dynamics by promoting the decay of *HAC1* RNA, suggesting a potential regulatory mechanism for linking vesicle trafficking to the UPR and ER homeostasis.

## Author Summary

The unfolded protein response (UPR), which allows eukaryotic cells to cope with stresses that impair their ability to properly fold and assemble their membrane and secreted proteins, is implicated in many human diseases such as diabetes, neurodegeneration, and cancer. In yeast, the *HAC1* gene encodes a transcription factor that plays a central role in regulating the UPR. By using protein microarrays to screen the yeast proteome, we discovered that Ypt1, a member of the Rab family of small regulatory GTPases, specifically interacts with the *HAC1* RNA. Further investigation revealed that Ypt1 associates with *HAC1* RNA under normal conditions, but not when the UPR is activated. The interaction with Ypt1 regulates the stability of *HAC1* RNA and plays a significant role in shaping the kinetics of the UPR. These findings provide new insight into a system with a critical role in defending cells against stress.

## Introduction

In eukaryotes, folding and assembly of most membrane-bound and secreted proteins takes place in the endoplasmic reticulum (ER). When proper folding of proteins in the ER is disrupted, cells turn on a protective mechanism known as the unfolded protein response (UPR). In a cascade conserved from yeast to humans, the UPR is activated through the ER-resident transmembrane kinase/endoribonuclease Ire1. In yeast, Ire1 cleaves the precursor to the RNA encoding the Hac1 transcription factor, *HAC1*u (‘u’ for ‘uninduced’), at the exon-intron junctions. In subsequent steps, the 5′ and 3′ terminal cleavage products, non-canonical exons, are ligated by the tRNA ligase Rlg1 to produce the mature RNA isoform, *HAC1*i (‘i’ for ‘induced’) [1], [2 and others]. Once translated, the Hac1 protein is translocated into the nucleus, where it activates the transcription of a set of genes encoding proteins important for alleviating ER stress [3].

To prevent UPR activation under normal conditions, the *HAC1* intron forms a stable secondary structure by base-pairing to the 5′UTR, rendering the unspliced RNA translationally inactive [4], [5]. This intron-5′UTR base-pairing, along with a conserved sequence in the 3′UTR, are both necessary, and together they are sufficient, for proper localization of *HAC1* RNA to the ER and for Ire1 cleavage during activation of the UPR [6]. Clearly, UPR activation is tightly regulated post-transcriptionally, but the non-canonical splicing of *HAC1* RNA may not be the only important control point. However, relatively few factors that interact with *HAC1* RNA have been identified.

We used an *in vitro* proteomic assay for RNA-binding to identify several novel *HAC1*-interacting proteins in *Saccharomyces cerevisiae*. The top *HAC1*-binding protein was Ypt1, an essential small Rab-family GTPase and a central regulator of ER-to-Golgi transport in the secretory pathway [7]. We further found that Ypt1 associates *in vivo* with unspliced *HAC1* in a UPR-dependent manner. *YPT1* knockdown resulted in elevated *HAC1* RNA levels under normal growth conditions, by reducing the rate of *HAC1* RNA decay. We found that normal Ypt1 expression was required for proper attenuation of the UPR upon recovery from ER stress. Extensive genetic interactions have previously established an important functional relationship between the UPR and vesicle trafficking pathways [8], [9], [10], [11]. Our results uncover a novel regulatory mechanism by which Ypt1, a key regulator of vesicle trafficking, controls the post-transcriptional fate of *HAC1*, the major transcription factor for the UPR, providing a regulatory link between these two critical pathways.

## Results

### *HAC1* RNA associates with small yeast GTPases *in vitro*

We recently described an unbiased approach to identify RNA-protein interactions *in vitro* on a genome-wide scale by binding RNA to protein microarrays that represent over 80% of the currently annotated *S. cerevisiae* proteome [12]. We used this strategy to screen for proteins that selectively bind to *HAC1* in preference to total yeast RNA (“Reference”). The greater the ratio of *HAC1*:Reference RNA fluorescent signal for a particular protein on the microarray, the greater the inferred specificity of that protein for *HAC1* relative to total RNA. To avoid potential biases associated with the fluorescent dye label, we performed multiple replicate experiments, swapping the dyes used to label the *HAC1* RNA and the Reference, respectively. We found five proteins with strong, consistent evidence of specific binding to *HAC1* RNA, based on significantly elevated Log_2_ *HAC1*:Reference ratios (*p*<10^−4^, combined *p*-values based on triplicate data; see “Materials and Methods” for details). This statistical threshold represents a stringent significance cutoff of *p*<10^−5^ (after correcting for multiple hypothesis testing) based on a null model of independent Gaussian distributions.

Two proteins, Rlg1 and Ire1, were already known to interact with *HAC1* (Table S1). Rlg1 was not represented on the microarrays we used in this study, and we did not detect any fluorescent signal from the Ire1 protein spots. We suspect that the batch purification procedure we used to prepare the protein microarrays [12] may have failed to isolate the transmembrane protein Ire1 in its functional form. The five proteins that reproducibly and specifically associated with *HAC1* RNA were Ypt1, Ypt7, Ypt32, Rho3 and Gis2 (Table S2). Of the five, only Gis2- a putative zinc-finger containing protein [13], had been reported to bind to RNA [12], [14], [15] (Table S1). We previously found that Gis2 associates with ∼150 RNAs *in vivo*, including *HAC1* RNA [12]. The remaining four proteins (the three Ypt proteins and Rho3), are small *ras*-family GTPases with roles in endo- and exocytosis [13] and no previous evidence for an RNA-binding function (Table S1).

These results add to increasing evidence that RNAs might use the cell's trafficking machinery for selective, targeted delivery to specific parts of the cell [12], [16], [17], [18], [19]. Such targeted localization could in turn provide a mechanism for linking an RNA's stability and translation to the activity of a cellular system. Ypt1, which associates with ER and Golgi membranes to control vectorial vesicle trafficking between the ER and the Golgi [20], [21], a process disrupted by ER stress, is a promising candidate regulator of *HAC1* expression in response to ER stress. We thus chose to test whether Ypt1 indeed interacts with *HAC1* RNA *in vivo* and if so, to investigate the nature and consequences of that interaction.

### Ypt1 associates with unspliced *HAC1* in the absence of ER stress

To identify RNAs associated with Ypt1 *in vivo*, we used a yeast strain expressing Ypt1 fused to glutathione S-transferase (GST) at its N-terminus, to avoid disrupting C-terminal prenylation, which is necessary for proper folding and function of the essential Ypt1 protein [22]. To confirm the functionality of this fusion protein, we transformed a yeast strain carrying the *ypt1-3* temperature-sensitive mutation, which displays a defect in ER-to-Golgi transport [20], [23], with the GST-Ypt1 plasmid. We found that even short-term induction of GST-Ypt1 expression (15 min) was sufficient to rescue the *ypt1-3* defect in carboxypeptidase Y (CPY) export from the ER (a marker for functional ER trafficking- [24]) (Figure S1a, lane 10 versus lane 5 for example). Because expression of the GST-fusion protein was induced by galactose, we also confirmed that induction of Ypt1 expression does not appreciably affect either abundance or splicing of *HAC1* RNA (Figure S1b and S1c).

**Figure 1:**
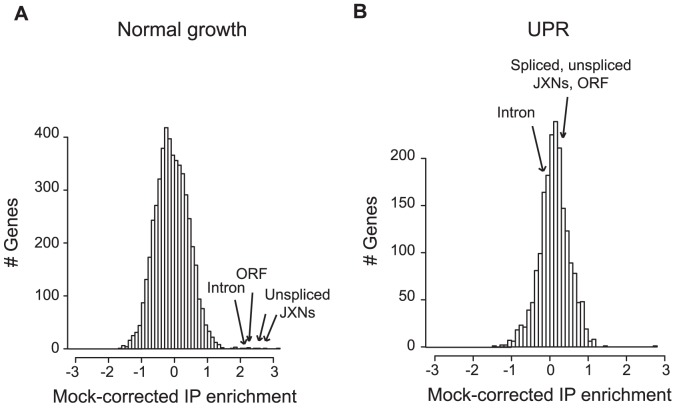
Ypt1 associates *in vivo* **with unspliced** ***HAC1*** **in a UPR–dependent manner.** GST-tagged Ypt1 was purified from yeast cells grown under normal (A) or UPR-induced conditions (B) using anti-GST conjugated beads. UPR was induced by addition of 10 mM DTT for 50 min. Co-purifying RNAs were labeled and bound to a DNA microarray. “Mock-corrected IP enrichment” values were calculated by subtracting Mock IP signal from Ypt1 IP signal on a gene-by-gene basis (see “Materials and Methods”). Arrows indicate enrichment values for *HAC1* probes (“JXNs” = junctions, 5 probes total present on the arrays- 2 unspliced junctions, intron, ORF, and spliced junction).

To test whether Ypt1 associated with specific RNAs *in vivo*, we affinity purified the tagged protein, then used DNA microarrays to identify any co-purifying transcripts [25]. “Mock” IPs done with lysates from the isogenic untagged parental strain provided a negative control. Under normal growth conditions, the RNA most reproducibly enriched in association with Ypt1 was the unspliced isoform of *HAC1* RNA (*HAC1*u) (Figure 1a, Table S3) (5-fold enrichment computed by SAM [26], *p* = 1.6×10^−4^ by one-tailed t-test). This result confirmed the interaction we had observed *in vitro* using protein microarrays. In the absence of ER stress, most *HAC1* RNA is unspliced [4]; the absence of detectable signal corresponding to the *HAC1* exon-exon junction thus still left open the possibility that Ypt1 might also bind to the spliced RNA. We therefore also evaluated the association under UPR-inducing conditions, in cells treated with DTT. To our surprise, the Ypt1-*HAC1*u association was lost under these stress conditions - a significant change from its behavior under normal growth conditions (∼6.5-fold change computed by SAM [26], *p* = 1.3×10^−2^ by one-tailed t-test) (Figure 1b, Table S3).

Thus, Ypt1 associates with unspliced *HAC1* RNA only in the absence of ER stress.

### Ypt1-*HAC1 in vivo* association requires two proteins implicated in UPR activation

How does this novel *in vivo* interaction relate to known mechanisms of *HAC1* RNA regulation? Specifically, we wondered if Ypt1 would associate with *HAC1* in cells defective in UPR activation. Ire1 and Ada5 are necessary for the initial processing of unspliced *HAC1* RNA upon ER stress induction. Ire1 is the nuclease that cleaves *HAC1* RNA, while Ada5, a component of the SAGA (Spt-Ada-Gcn5-acetyltransferase) histone acetylation complex [27], interacts directly with Ire1 and is also required for the proper splicing of *HAC1*i [28] (Table S1). We investigated if deleting either protein would have an effect on the Ypt1-*HAC1* interaction by purifying Ypt1 from *ire1*Δ or *ada5*Δ cells. In contrast to the results seen in wild-type cells, we found no evidence for enrichment of *HAC1* RNA in association with Ypt1 in these mutant cells. The absence of *HAC1* RNA enrichment in each mutant was in significant contrast to the enrichment observed in wild-type cells: ∼18-fold less (*p* = <4.7×10^−3^) or ∼11-fold less (*p* = 1.6×10^−2^) in *ire1*Δ and *ada5Δ* cells, respectively (*p*-values computed by SAM [26]) - Table S4.

Loss of the Ypt1-*HAC1* interaction in *ire1*Δ and *ada5*Δ strains let us to consider if the two proteins physically interact with Ypt1 and/or *HAC1*. We tested if Ypt1 bound Ire1 or Ada5 by immunopurification of Ypt1 and Western blotting for Ire1 or Ada5 (see “Materials and Methods”). In formaldehyde-crosslinked cells, tagged Ypt1 was associated with Ada5, but not with Ire1 (Figure S2b). The Ada5-Ypt1 association was also detectable, albeit to a considerably lesser extent in the absence of crosslinking (Figure S2c). This raised the possibility that Ada5 could be a component of the Ypt1-*HAC1* complex. However, when we purified Ada5 protein from cells and tested for interaction with *HAC1* RNA, we found no significant *HAC1* enrichment (Table S5). Our results, therefore, are inconsistent with Ada5 or Ire1 stably associating with Ypt1-bound *HAC1*. This suggests that Ire1 and Ada5 may function to recruit Ypt1 and *HAC1* RNA in proximity to each other, and this recruitment may be required for proper formation of the GTPase-RNA complex (see “Discussion” section).

### Ypt1 regulates *HAC1* transcript levels

**Figure 2:**
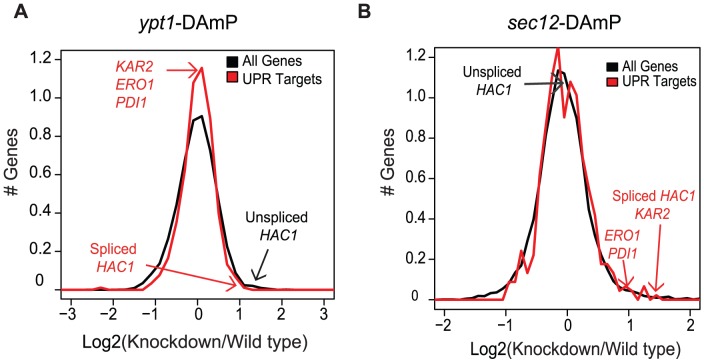
Ypt1 knockdown affects *HAC1* **splicing and expression.** (A) Gene expression of *ypt1* (A) or *sec12* (B) knockdown strain compared to isogenic parental wild type strain. In black, the signal distribution of (Log_2_(Knockdown/WT)) values for all genes is shown. In red, the distribution of (Log_2_(Knockdown/WT)) values for annotated UPR target genes [3] is shown. Red arrows point to values for canonical UPR target genes (*HAC1*i, *KAR2*, *ERO1*, *PDI1*) and black arrows show values obtained from *HAC1*u probes. Data presented are averages of two replicate experiments.

What role, if any, does the association between Ypt1 and *HAC1* RNA play in regulating *HAC1* expression? *YPT1* is an essential gene [7]. We therefore used a strain from the yeast hypomorphic allele (DAmP) library to determine whether reduced expression of *YPT1* has any effect on *HAC1* RNA levels [29]. We compared global RNA expression patterns in *ypt1*-DAmP and wild type strains using DNA microarrays. As expected, *YPT1* transcript levels were significantly reduced in the *ypt1*-DAmP mutant (∼2.5-fold reduction, *p* = 7.4×10^−8^ by t-test), confirming successful knockdown (Table S6). Notably, levels of both unspliced and spliced *HAC1* transcripts were significantly higher (∼2-fold increase, *p* = 1.2×10^−6^ by t-test) in the knockdown strain, pointing to a functional role for Ypt1 in regulation of *HAC1* (Figure 2a, Table S6). In the absence of ER stress, however, this increase in spliced *HAC1* was not sufficient to induce the UPR, as reflected by the expression of key UPR target genes relative to all genes (*p* = 0.1 by t-test). This could be explained if the *HAC1*i mRNA is not translated as efficiently under these circumstances, or if the overall increase in *HAC1*i levels is unequally shared among the different cells in the population, such that induction of UPR targets in a subset of cells is obscured by the absence of UPR activation in the rest of the population. Alternatively, the ∼2-fold increase in *HAC1*i expression may not be enough to surpass a threshold level for eliciting UPR.

Increased *HAC1* transcript levels in the *ypt1*-DAmP cells could reflect a direct effect of Ypt1 on expression of the *HAC1* RNA or an indirect effect: impaired export of proteins from the ER, leading to activation of the UPR. Indeed, when we examined the ability of CPY to transit from the ER to the vacuole by Western blotting, we found a higher fraction of CPY precursor in *ypt1*-DAmP compared to wild type cells (Figure S3, lanes 1 and 3), a hallmark of defect in ER export. In order to assess the possibility of indirect effect of trafficking block on *HAC1* RNA levels, we compared global RNA expression patterns in *ypt1*-DAmP and another ER export mutant, *sec12*-DAmP. Sec12 is a guanine nucleotide exchange factor, required for COPII vesicle formation at the ER [30], [31] (Table S1). Similar to Ypt1, intact Sec12 is essential for proper ER-to-Golgi transport [30], which we verified by measuring CPY precursor accumulation in *sec12*-DAmP mutant cells (Figure S3, lane 2). The *sec12*-knockdown strain exhibited a more severe block in ER export (Figure S3, compare lanes 1 and 2) that also led to UPR activation (Table S6 and Figure 2b, *p* = 1.1×10^−2^ by t-test). However, in contrast to their significant elevation in *ypt1*-DAmP strains, levels of *HAC1*u remained unchanged in *sec12*-DAmP cells (Figure 2b, *p* = 0.1 by t-test). Thus, the difference in phenotype between the two DAmP strains suggests that the changes in *HAC1*u/i expression in the *ypt1*-DAmP strain were due specifically to impairing the Ypt1-*HAC1* association.

### Ypt1 regulates *HAC1* RNA expression by promoting RNA decay of unspliced *HAC1*

**Figure 3:**
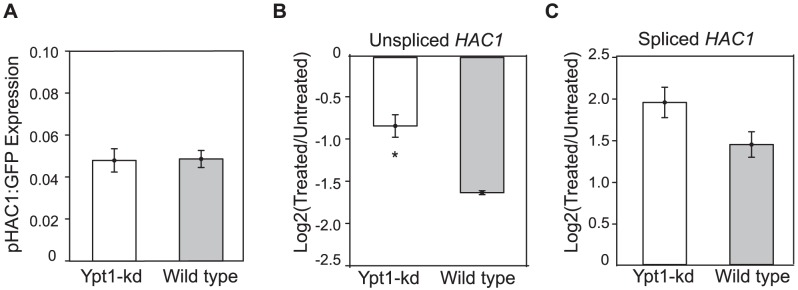
Ypt1 knockdown effect on *HAC1* **RNA transcription and decay.** (A) Knocking down *YPT1* does not affect *HAC1* promoter activity as evaluated by assaying RNA levels of a transcriptional reporter gene containing the *HAC1* promoter sequence fused to a *GFP* ORF sequence and the *ACT1* 3′UTR. Normalized expression, reported as a fraction of actin RNA levels, was determined using quantitative RT-PCR. Data represent the average of three replicate experiments. (B-C) Quantitative RT-PCR measurements of unspliced (B) and spliced (C) *HAC1* RNA expression 30 min post addition of 3 ug/ml thiolutin (“Treated”) relative to *HAC1* RNA levels in untreated cells. Values are normalized to GAPDH levels and data are averages of 3–4 replicates. Asterisks indicate significant differences. * = *p*<0.05 by t-test.

Our next goal was to determine what accounts for the effect of Ypt1 on *HAC1* transcript levels. Elevated *HAC1* RNA levels observed in the *ypt1*-knockdown strain could stem from an increase in transcription, enhanced RNA stability, or both. Previous reports have shown that *HAC1* transcription is positively autoregulated by the Hac1 protein: Hac1p binds to UPR elements (UPRE) present in its own promoter [32]. Despite the relatively elevated levels of *HAC1* RNA, the basal concentration of Hac1p was not increased in the *ypt1*-DAmP strain (*p* = 0.4 by t-test) (Figure S4), consistent with the lack of UPR induction under normal conditions in the knockdown (Figure 2). It is still conceivable that *HAC1* transcription could be affected by Ypt1-dependent factors other than Hac1p itself. Indeed, independent mechanisms allow restricted cell survival under ER stress in mutants lacking *HAC1* UPRE sites [32]. Thus, a Hac1p-independent change in promoter activity was still a feasible explanation for the difference in RNA expression in the *YPT1* mutant strain. To measure the activity of the *HAC1* promoter, we generated a *HAC1* transcription reporter gene by fusing the *HAC1* promoter region to the green fluorescent protein (GFP) coding sequence. We did not observe significant differences in the amounts of *GFP* RNA produced between *ypt1*-DAmP and wild type (*p* = 0.9 by t-test) - Figure 3a. Although we cannot exclude the possibility that *HAC1* transcription may be influenced by intragenic or distal regulatory elements not present in the construct, the data suggest that increased transcription is unlikely to account for increased *HAC1*u abundance in the mutant strain.

Another possibility to explain the overall increase in *HAC1*u expression was an altered decay rate. To evaluate RNA stability, we treated mutant and wild type cells with thiolutin to inhibit transcription [33], , and measured RNA levels before and after drug addition by quantitative RT-PCR. We estimated half-life by comparing *HAC1*u levels after 30 min treatment with thiolutin to its pre-treatment abundance. We found that both unspliced and spliced *HAC1* isoforms decayed more slowly in the *YPT1* mutant (Figure 3b and 3c). We estimated the half-life of *HAC1*u in wild type cells to be 19 min, consistent with previous reports [10], [35]. In the DAmP strain, however, the rate of *HAC1*u RNA decay was markedly reduced (*p* = 1.7×10^−2^ by one-tailed t-test) to an estimated half-life of 37 min. This two-fold difference in *HAC1*u RNA stability is sufficient to account for the ∼2-fold difference we had measured in steady-state RNA expression levels (Table S6). Thiolutin treatment triggers ER stress, which probably accounts for the increase in *HAC1*i levels following drug addition in both the wild type and *ypt1*-DAmP strains (Figure 3c).

If Ypt1 normally promotes *HAC1* RNA decay, abolishing the Ypt1-*HAC1* interaction should impair *HAC1* RNA stability. Since we found that the *HAC1* RNA-Ypt1 association was lost in *ire1Δ* and *ada5Δ* strains (Table S4), *HAC1* RNA might be expected to be more stable in these mutants. Indeed, based on assaying decay following thiolutin treatment, we found that the half-life of *HAC1* RNA was 1.6 and 2.7 times longer in the *ada5Δ* and *ire1Δ* strains, respectively, than in the corresponding wild-type strain (*p* = 5.0×10^−2^ and 1.3×10^−2^ by t-test, respectively) - Figure S5.

These results imply that Ypt1 protein controls *HAC1* expression by accelerating the decay of *HAC1* RNA.

### Ypt1 is required for normal UPR signaling dynamics

To begin to assess the physiological significance of the Ypt1-*HAC1* interaction, we evaluated the effect of a partial loss of Ypt1 on the dynamics of the ER stress response. First, we tested whether the *ypt1*-DAmP mutation impaired growth in the presence of tunicamycin (a drug that impairs glycosylation and thus proper protein folding in the ER). We found no appreciable growth defect in the mutant compared to wild type (Figure S6a). To more sensitively evaluate the potential role of Ypt1 in the dynamics of the UPR, we tracked the mRNA dynamics of four canonical UPR targets - *KAR2*, *ERO1*, *PDI1* and *HAC1*i (Table S1) - in DTT-treated cells, by quantitative RT-PCR. The levels of *KAR2*, *ERO1* and *PDI1* did not differ significantly between the *YPT1* mutant and wild type cells in the first 20 min after DTT addition (Figure S6b–S6d). The expression of spliced *HAC1* RNA, however, was 1.7-fold higher in the DAmP strain than in the wild-type cells after 20 min of DTT exposure (*p* = 4.1×10^−3^ by t-test) (Figure S6e). Hac1 protein levels as well were ∼1.5 times higher in the mutant compared to wild-type cells after 20 min of DTT exposure (*p* = 6×10^−2^ by t-test), based on quantitative Western blot analysis (Figure S6f). Thus, although the *HAC1* RNA-Ypt1 association did not appear to impair initiation of the UPR (up to 10 min), the later phases (beyond 10 min after UPR induction) - possibly including the kinetics of recovery - differed between mutant and wild type.

**Figure 4:**
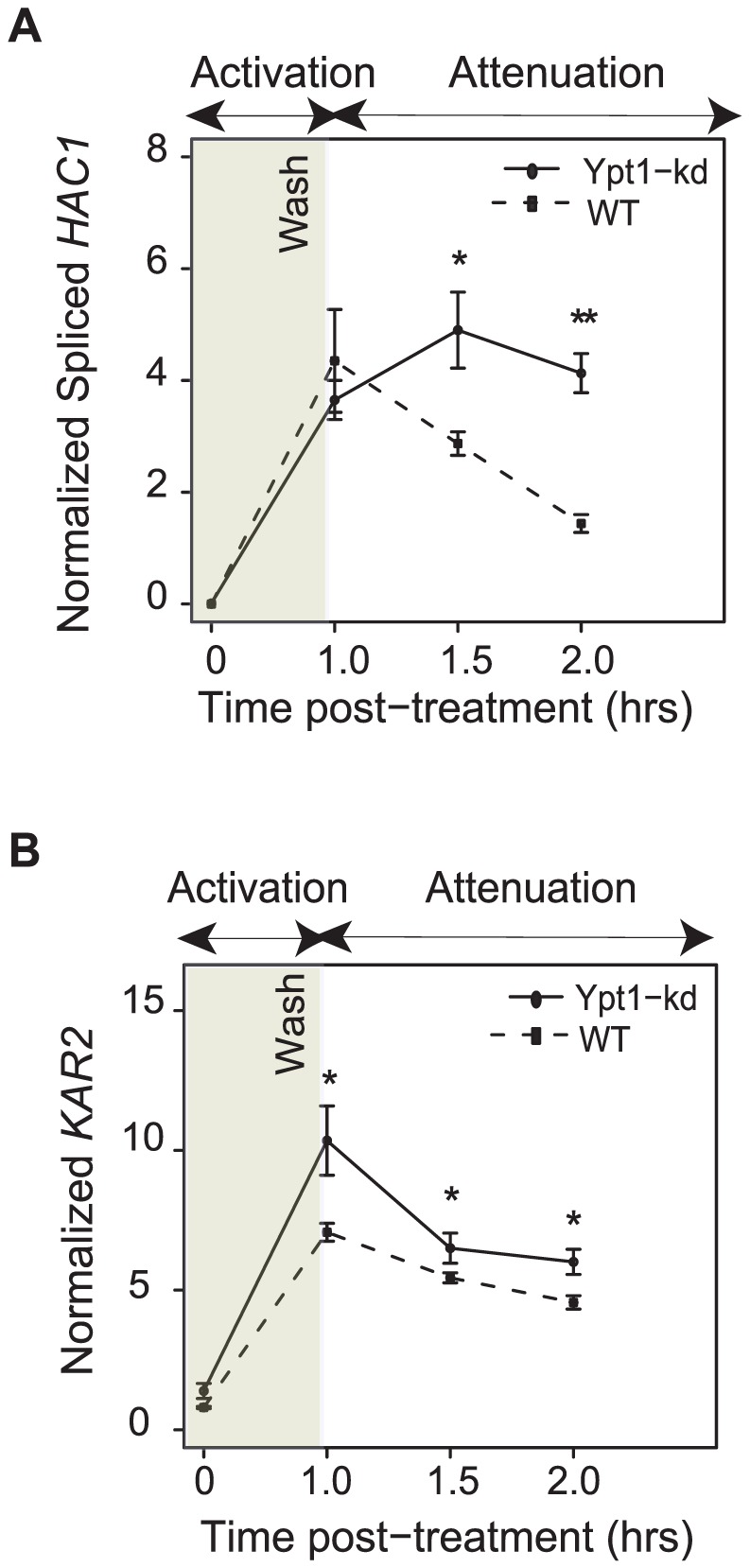
Ypt1 levels affect UPR attenuation. *ypt1*-DAmP or its isogenic parental wild type strain were treated with 10 mM DTT for 1 hr, then washed, and re-suspended in fresh YPD media. A recovery assay was performed (up to one hour after wash) and expression levels of spliced *HAC1* (A) and the canonical UPR target gene *KAR2* (B) were measured by quantitative RT-PCR. Data represent average of four replicates per strain. All values are normalized to actin levels. Asterisks indicate significant differences. * = *p*<0.05, ** = *p*<0.005 by t-test.

To examine the role of Ypt1 in attenuating the ER stress response, we induced UPR with DTT for one hour in Ypt1 knockdown or wild-type cells, respectively, then transferred cells to fresh media and monitored levels of *HAC1*i and *KAR2* RNA for another hour by quantitative RT-PCR. Transcripts of both genes persisted at significantly higher levels in the *ypt1*-knockdown strain than in the wild type cells - *HAC1*i abundance levels were ∼3 times higher (*p* = 2.2×10^−5^ by t-test) and those of *KAR2* RNA were ∼1.3 times higher (*p* = 9.0×10^−3^ by t-test) in the mutant, an hour after transfer to non-inducing media (Figure 4a and 4b, respectively). By 3 hours after removal of DTT, expression of *HAC1*i had returned to near-basal levels in both wild-type and mutant cells (Figure S7).

We speculate that delayed attenuation of the UPR in y*pt1*-knockdown cells (Figure 4) could point to a key role played by Ypt1 in aiding cellular recovery from ER stress.

## Discussion

UPR activation is a tightly regulated process [6], [36], [37], in which recruitment of *HAC1* RNA to the ER followed by non-canonical splicing/ligation [1], [38], [39] is required for proper cascade initiation. We identified five proteins that specifically associated with *HAC1* RNA *in vitro* by a proteomic assay. Other proteins that consistently ranked highly for *HAC1* RNA association in this assay (Table S2), but that we have not yet investigated further, may also have functionally significant interactions with *HAC1* RNA. Remarkably, three of the five proteins we found to interact with *HAC1* RNA were *ras*-superfamily GTPases with roles in endocytosis and exocytosis [40], [41], [42], [43]; none had previously been implicated as components of RNA-protein complexes. Recent reports show that a number of enzymes, a significant fraction of which participate in vesicle trafficking, have “moonlighting” roles as RBPs [12], [15], [44], [45], [46], [47]. In light of these findings, the possibility that Rab GTPases might moonlight as RBPs to regulate *HAC1* is intriguing. As we focused the present study on Ypt1, it remains to be determined whether and how Ypt7 and Ypt32 (Table S2) might affect *HAC1* expression.

We confirmed that the *in vitro* binding screen reflected an *in vivo* association between Ypt1 and *HAC1* RNA. The Ypt1-*HAC1* interaction was disrupted by conditions that trigger ER stress (Figure 1). The interaction had functional consequences– knocking down expression of Ypt1 led to reduced *HAC1* RNA decay and, consequently, higher *HAC1* RNA expression levels (Figure 2 and Figure 3). Moreover, we found that UPR kinetics were distinctly abnormal in *YPT1*-deficient cells (Figure 4), establishing a physiologically significant role for Ypt1 in the regulation of this critical stress response.

Even though Ypt1 consistently and significantly interacted with *HAC1* RNA both *in vitro* and *in vivo*, it remains possible that the Ypt1-*HAC1* interaction could be indirect. Even in the *in vitro* protein microarray experiment, a distinct *HAC1* RNA-binding protein could have remained associated with Ypt1 in the high-throughput purification procedure used to prepare the protein microarrays [12]. The *in vivo* IPs required the use of chemical crosslinking, which might preserve protein complexes responsible for the association. We evaluated the possibility that Ire1 or Ada5 could be RBP adaptors present in the Ypt1-*HAC1* complex. However, Ire1 did not physically associate with Ypt1 (Figure S2), and Ada5 did not interact with *HAC1* RNA (Table S5). It is also possible that the Ypt1-*HAC1* interaction is direct, but weak and/or transient. For example, complex formation with *HAC1* RNA could be localization-dependent (see below). Because Ypt1 can toggle between two nucleotide states- GDP or GTP-bound, it is feasible that only one of these conformations binds RNA. A direct but transient interaction, and/or one that is nucleotide- or localization-dependent could be hard to detect reproducibly in the absence of crosslinking.

**Figure 5:**
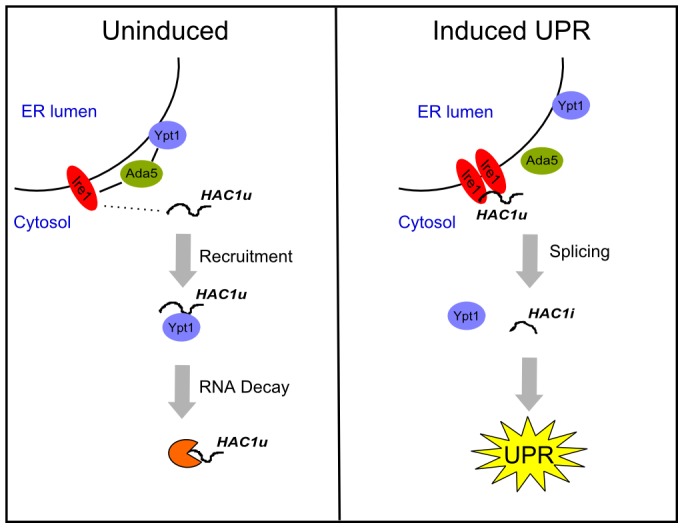
Ypt1 negatively regulates *HAC1*u RNA expression in the absence of ER stress. Under normal growth conditions (left panel). Ypt1 regulates *HAC1* RNA expression by destabilizing the transcript. This is likely accomplished by recruiting *HAC1* away from the ER and in proximity to decay factors. Ire1 and Ada5 are necessary for the interaction and could play an active role in recruiting *HAC1* to the ER, thus facilitating the Ypt1-*HAC1* association. Under UPR-induced conditions (right panel). The GTPase no longer interacts with *HAC1*. Instead, *HAC1*u is spliced by Ire1 and the mature *HAC1*i mRNA is translated to activate the ER stress response cascade. Dotted line denotes putative recruitment of *HAC1*u by Ire1; solid lines show established interactions. *HAC1*u = unspliced *HAC1*; *HAC1*i = spliced *HAC1*; ER = endoplasmic reticulum; UPR = unfolded protein response.

Given the importance of subcellular localization to the functions of both Ypt1 and *HAC1* RNA, there are compelling reasons to consider whether their interaction may occur specifically at the ER. First, previous reports have shown that a substantial fraction of Ypt1 is localized on the ER membrane [20]. Second, we found that an intact *HAC1* 3′UTR, which is required for proper ER localization [6], is also necessary (Table S4) but not sufficient (data not shown) for the Ypt1-*HAC1* interaction. Last, our data establish that *IRE1* and *ADA5* are essential for the association. Ada5 physically binds to Ire1 [28], and Ire1 is an integral ER protein that is necessary for proper ER-localization of *HAC1* RNA [6], [28]. Therefore, we propose that Ire1 may recruit unspliced *HAC1* RNA to the ER, and Ada5 may recruit Ypt1 in proximity to Ire1, thus enabling Ypt1-binding to *HAC1* (Figure 5).

What characteristics of Ypt1 make it suitable for its newly identified role in UPR regulation? An association between *HAC1*u and ER-Golgi transport vesicles (and perhaps also transport vesicles further downstream in the secretory pathway) could provide an efficient way to recruit the *HAC1* RNA away from the ER-localized UPR splicing machinery in the absence of stress (Figure 5). Since Ypt1 orchestrates multiple steps of ER-to-Golgi transport, including budding of ER vesicles [48] and their subsequent fusion with the Golgi [20], [23], as well as generation of vesicles at the Golgi and their docking at the ER [49], it would be a natural candidate for modulating a potential interaction between *HAC1* and ER-Golgi transport vesicles to divert any ER-proximal *HAC1* RNA away from Ire1 during normal growth (Figure 5). The idea of the vesicle machinery playing a role in RNA localization is not far-fetched: studies done in two diverse systems have implicated the Rab11 GTPase- important for recycling of cell surface proteins [18], [50]- in RNA localization, and anchoring [16], [17], [19].

Ypt1-dependent regulation of *HAC1* RNA stability establishes a regulatory link that may underlie known functional relationships between the UPR and vesicle trafficking pathways. Past studies have shown that defects in the secretory pathway induce the UPR and that a functional UPR is required for cell survival under these conditions [8], [10]. Furthermore, constitutive activation of the UPR can rescue growth defects of vesicle trafficking mutants [8], [9], [11]. These important findings have been largely interpreted as reflecting a reactive process, whereby disruption of ER export causes accumulation of unfolded proteins, which in turn triggers the UPR [11]. Our results raise the possibility that deficiencies in ER trafficking may also interfere with Ypt1-mediated control of *HAC1* expression, thus potentiating the UPR via a proactive regulatory mechanism. Ultimately, active regulatory events that enable communication between the UPR and vesicle trafficking pathways may contribute to proper cellular homeostasis in response to ER stress.

The present study describes a novel mode of post-transcriptional regulation of the *HAC1* RNA through association with a Rab-family GTPase. The regulatory logic and molecular mechanisms of Ypt1-dependent decay and the specific role of each identified component (including Ada5, Ire1, Ypt1, and *HAC1*) in mediating crosstalk between the UPR and vesicle trafficking systems are new avenues for further investigation.

## Materials and Methods

### Yeast strains and microarray data

Yeast strains and their corresponding genotypes are listed in Table S1. All commercially available *Saccharomyces cerevisiae* strains (GST-tagged, knockdowns and knockouts) were purchased from Open Biosystems. The *ypt1-3* and the corresponding wild-type strains were a gift from Charles Barlowe (Hanover, NH). Lithium acetate transformations were performed according to standard protocols [51]. For generating strains used in experiments testing *ADA5* and *IRE1* requirement, *GST-YPT1* plasmid was isolated from the Open Biosystems strain and transformed into *ire1*Δ or *ada5*Δ. For experiments testing *HAC1* promoter activity, the ∼1000 bases upstream of the annotated start site were fused to the *GFP* coding sequence and the *ACT1* 3′UTR and ligated into pRS315 [52]. For experiments measuring *HAC1* decay in knockout mutants, *HAC1* splice junction mutant (*HAC1-G1137C*) [53] was ligated to plasmid pRS406 and integrated into the Ura locus of *ada5Δ*, *ire1Δ*, and *hac1Δ* strains.

For Ire1-Ypt1 and Ada5-Ypt1 co-purification experiments, we initially wished to use the commercially available TAP-tagged strains, but found that, consistent with previous observations [6], Ire1 did not tolerate a large tag on its C-terminus (data not shown). Therefore, we generated HA-tagged strains for the proteins (*IRE1, ADA5, MRS6, PUF5*). The 3xHA tag sequence was PCR-amplified from the pYM1 plasmid [54] and integrated into each corresponding locus. Strains were subsequently transformed with the GST-*YPT1* plasmid. HA-tagged Ire1 was functional (Figure S2a).

All microarrays were scanned with GenePix Pro 6.0 (Molecular Devices). Data were deposited on Stanford Microarray Database (http://smd.stanford.edu/) and GEO (www.ncbi.nlm.nih.gov/geo) under accession number GSE33751.

### Protein microarray experiments

Samples were prepared and binding reactions were performed as described in [12]. Full length HAC1 RNA, including UTRs (annotated by [61]) and intron, was transcribed *in vitro* using the Epicentre AmpliScribe T7 Aminoallyl-RNA Transcription Kit (#AA5025) and then Cy-dye labeled (GE Healthcare, Cat# RPN5661). Total yeast mRNA labeled with a different Cy-dye was used as a “Reference” [12]. All RNA samples were refolded by incubating at 70°C for 5 min and then cooling on ice prior to protein microarray binding. For each protein, a mean-normalized Log_2_ (*HAC1*/Total mRNA) ratio was calculated in order to identify specific *HAC1*-interactors. Six replicate experiments were performed, including three replicates using Cy5-labeled *HAC1* RNA vs Cy3-labeled Reference RNA, and three additional “dye-swap” replicates with Cy3-*HAC1* vs Cy5-Reference. For each experiment, proteins that failed to give detectable fluorescent signal above 1.5 times the background in either the Cy5 or Cy3 channels were filtered out and excluded from further analysis. Each Log_2_ ratio for the remaining proteins was converted into a ‘*z*-score’ by subtracting the mean value and dividing by the standard deviation. The computed *z*-scores were used to calculate *p*-values reflecting the significance of the specific interaction with *HAC1* RNA for each protein based on the Gaussian distribution. For each set of “dye-swap” experiments, a single combined *p*-value was computed as the product of the *p*-values for its three replicates. Proteins with combined *p*-values satisfying *p*<10^−4^ for both sets of dye-swap experiments were classified as high-confidence *HAC1* interactors (Table S2). We acknowledge that proteins that did not meet our stringent threshold criteria for specific *HAC1*-binding, but still ranked at the top in Table S2, may also represent real *HAC1*-interacting proteins.

### *In vivo* immunopurification of GST-Ypt1 and analysis of targets

Open Biosystems haploid yeast strain containing GST-tagged Ypt1 was grown first in SC-Ura overnight and then diluted into SC-Ura/2% raffinose. Cells were grown to OD_600_∼0.4 and protein expression was induced with 4% galactose for 2 cell divisions. Formaldehyde was added for the last 5 min to a final concentration of 1%. For experiments, in which UPR was induced, DTT was added to a final concentration of 10 mM for the last 50 min of growth, while formaldehyde was added for the last 5 min. Cells were washed twice with Buffer A (50 mM Tris pH 7.4, 140 mM NaCl, 1.8 mM MgCl2, 0.1% NP-40) and lysed using a Cryogenic grinder (Retsch). Lysed cells were resuspended in Buffer B (Buffer A supplemented with 0.5 mM DTT, 40 U/ml RNase Inhibitor, 1 mM PMSF, 0.2 mg/mL heparin, protease inhibitor complete tablet from Roche) and sonicated. Lysates were cleared by centrifugation for 10 min at 8,000 rpm/4°C.

Anti-rabbit Dynabeads M-280 from Invitrogen (Cat# 11203D) were prepared by washing three times for 3 min each at RT with 1 volume 1X PBS/0.1% BSA. Beads were resuspended in 1 volume 1X PBS/0.1% BSA. Rabbit anti-GST antibody from Open Biosystems (Cat# CAB4169) was added at a ratio 100 ug antibody per 10 mgs beads and incubated overnight at 4°C on a rotator. Excess antibody was removed by washing with 1 volume 1X PBS/0.1% BSA three times for 15 min each at 4°C. Beads were resuspended to original volume in Buffer B.

Lysates were incubated with 500 ul anti-GST conjugated beads per 1L original cells for 2 hrs at 4°C on a rotator. A fraction (≤300 ul) of the depleted supernatant served as a reference (see below). We took advantage of the formaldehyde crosslinking, which allows for more stringent washes. Different salt and detergent concentrations were tested to find optimal conditions that would minimize indirect interactors without affecting GST tag folding. Beads were washed twice in 1.5 ml Buffer B for 10 min/4°C/rotator each, then once in 1.5 ml “high salt” (2M NaCl) for 10 min/4°C/rotator, once in 1.5 ml “high detergent” (2M urea) for 10 min/4°C/rotator, and finally twice in 1.5 ml Buffer C (50 mM Tris pH 7.4, 140 mM NaCl, 1.8 mM MgCl2, 0.01% NP-40, 10% glycerol, 1 mM PMSF, 0.5 mM DTT, 40 U/ml RNase Inhibitor, protease inhibitor complete tablet) for 10 min/4°C/rotator each. After washing, beads were resuspended in 300 ul Buffer C supplemented with 1% final SDS and heated at 70°C for 45 min with constant mixing to de-crosslink samples and elute antibody-bound protein-RNA complexes from the beads. Cells for “Mock IPs” from isogenic parental untagged strain BY4741 were grown in synthetic media with 2% raffinose, treated with galactose, crosslinked, lysed, and incubated with anti-GST beads following the same procedure. The same protocol was used for all other GST-Ypt1 containing strains and respective mocks.

RNA from both depleted supernatant (“Reference”) and eluted fraction (“IP”) was isolated using Purelink RNA Mini Kit from Invitrogen (Cat # 12183018A). Up to 5 ug of RNA were amplified with Ambion's Aminoallyl MessageAmp II aRNA Kit (Cat# AM1751 or AM1819) and labeled with Cy5 (“IP”) or Cy3 (“Reference”) according to manufacturer recommendations (GE Healthcare, Cat# RPN5661). Samples were prepared and bound to oligonucleotide microarrays as described previously [55].

For growth under normal conditions (i.e. in the absence of UPR), we performed a total of 6 GST-Ypt1 IPs and 6 corresponding Mocks (total 12). Data were median-centered on an array-by-array basis and “Log_2_Ypt1-IP Enrichment” for each gene was calculated as:
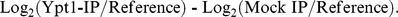
We filtered for genes that had signal 1.5 times greater than background in either Cy3 or Cy5 channel in ≥9 of 12 experiments and ranked genes based on their “Ypt1 Enrichment” signal. Probes spanning the unspliced isoform of *HAC1* gave consistently high signal with *p* = 1.6×10^−4^ by unpaired t-test (Table S3).

For UPR experiments and IPs with knockout strains, 2 replicates of each Ypt1 IP and Mock IP were performed. “Log_2_Ypt1-IP Enrichment” was calculated as described above for genes that had signal in >50% of the replicates. *p*-values for under-enrichment in the UPR IP were obtained by one-tailed t-test comparing “Log_2_Ypt1-IP Enrichment” values for the *HAC1* probes in the UPR to the normal IPs. *p*-values for under-enrichment for mutant strains were calculated based on FDR values from pairwise SAM analysis [26] of wild-type versus each of the knockout IPs.

### *YPT1* knockdown gene expression profiling

For experiments testing growth phenotype on tunicamycin plates, dilution series of *ypt1*-DAmP [29], *ire1*Δ and BY4741 strains were plated on YPD or YPD+0.5 ug/ml TM according to standard plating assay protocols.

For UPR induction time course experiments, *ypt1*-DAmP strain and isogenic wild-type BY4741 strain, respectively, were grown in YPD to OD_600_∼0.7. A sample was taken out (“uninduced”), and DTT was added to a final concentration of 10 mM to the remaining cells. Samples were taken out at 5, 10, and 20 min, cells were collected by vacuum filtration and quick-frozen. RNA was isolated with hot phenol [56] and reverse transcribed with a mix of oligo(dT) and a random nine-mer primer. qPCR was performed with primers for *KAR2*, *ERO1*, *PDI1*, spliced *HAC1*, and *ACT1* (normalization control). Experiments were performed in duplicate, actin-normalized data were averaged for each strain, and one-tailed unpaired t-test analysis was performed to compare the measurements at each time-point between strains.

For UPR attenuation time course experiments, *ypt1*-DAmP strain and isogenic wild-type BY4741 strain were grown in YPD to OD_600_∼0.7. A sample was taken out (“uninduced”), and 10 mM DTT was added to remaining culture. One hour after induction, a second sample was taken out (“pre-wash”), and the rest of the culture was re-suspended in fresh YPD media. Samples were collected at indicated times, RNA was extracted with hot phenol and qPCR was performed and analyzed analogous to UPR induction experiments (see above). Experiments were done in quadruplicate for each strain.

For *ypt1*-DAmP and *sec12*-DAmP experiments, 50 ml of each DAmP and BY4741 were grown in YPD to OD_600_∼0.7. Cells were collected, lysed and RNA was extracted with hot phenol. Samples for microarray analysis were prepared as described before [55] using 30 ug total RNA as starting material. “Uninduced” DAmP cDNA was labeled with Cy5 and “uninduced” BY4741 cDNA was labeled with Cy3.

### *YPT1* knockdown protein determination

*YPT1* mutant and parental BY4741 cells grown to OD_600_∼0.7 in duplicate. For quantifications done under normal condition, a portion of the cells was harvested by centrifugation prior to drug addition. For UPR expreriments, the remaining cells at OD_600_∼0.7 were treated with 10 mM DTT for 20 min and harvested by centrifugation. Lysates were boiled in 4X Sample Buffer (Biorad, Cat# 161-0791) and loaded on a gel. Anti-Hac1 antibody, a generous gift from Dr. Peter Walter (San Francisco, CA), and anti-Gapdh (Abcam, Cat# ab93378) were used at 1∶2,000 dilutions. Staining was done overnight at 4°C and appropriate secondary HRP-conjugated antibodies were used to detect protein levels. ImageJ [57] was used for precise quantification; *p*-values were determined by one-tailed unpaired t-test.

### *HAC1* promoter activity in *YPT1* knockdown

To evaluate potential effects of knocking down Ypt1 on *HAC1* transcription, *ypt1*-DAmP or isogenic BY4741 wild type were transformed with pRS315 bearing *GFP* under the control of the *HAC1* promoter (see “*Yeast strains*” above). Cells were grown in YPD to OD_600_ = 0.6–0.7 and harvested. Total RNA was extracted with hot phenol [56] and reverse transcribed with a mix of oligo(dT) and a random nine-mer primer. qPCR was performed with primers for *GFP* or *ACT1* (control). Experiments were performed in triplicate, actin-normalized data were averaged for each strain, and unpaired t-test analysis was performed to compare the measurements between strains.

### Decay measurements

Immediately prior to drug addition, samples were removed (“Untreated”). Thiolutin (from fresh 1 mg/ml stock in DMSO) was added to 3 ug/ml final concentration to cells at OD_600_∼0.8 for 30 min (“Treated”). Cells were collected by rapid filtration and RNA was extracted with hot phenol. Samples for quantitative RT-PCR were prepared by reverse transcription of total RNA with oligo(dT)/random nine-mer primer mix followed by qPCR with Taqman probe specific for spliced *HAC1* or primers recognizing the unspliced *HAC1* isoform. *GAPDH* levels were used to normalize data and unpaired t-test analysis was performed to compare the measurements. RNA half-lives were determined with the formula: Half-life = (t1−t0)/Log_2_ (X(t1)/X(t0)), where X(t) is the expression level at time t following thiolutin treatment.

Since *HAC1* splicing is abolished in the *ADA5* and *IRE1* knockout mutants [6], [28], we generated *ada5Δ*, *ire1Δ*, and *hac1Δ* strains with integrated “unspliceable” *HAC1* variant (*HAC1-G1137C*) (see “*Yeast strains*” subsection above and Table S1). The purpose was to rule out any non-specific effects of *HAC1* splicing and UPR induction post-thiolutin treatment on *HAC1* stability.

## Supporting Information

Figure S1 GST-Ypt1 expression rescues *ypt1-3* temperature-sensitive mutant phenotype and does not affect significantly *HAC1* RNA levels or splicing. (A) Galactose-induced expression of GST-Ypt1 rescues *ypt1-3* temperature-sensitive mutant phenotype. Cells were grown at room temperature to mid-log phase, then either moved to 37°C or left at 25°C for 4 hours. CPY-precursor accumulates at restrictive (37°C) relative to permissive (25°C) temperature in *ypt1-3* (lane 5 compared to lane 1; an isogenic wild-type strain serves as a control- lanes 6 versus 2). Anti-CPY (Invitrogen) was used at 1∶2,000 dilution. GST-Ypt1 was transformed into *ypt1-3* or wild-type and expression was induced for indicated times (lanes 7–10 for *ypt1-3*/GST-Ypt1 induction for 0.25, 0.5, 1 or 2 hrs and lanes 11–13 for wild-type/GST-Ypt1 induction for 0.5, 1 or 2 hrs). GST-Ypt1 expression was verified by re-probing with anti-GST antibody (data not shown). (B–C) Induced expression of GST-Ypt1 does not lead to significant changes in *HAC1* RNA levels (B) or splicing (C). Quantitative RT-PCR assay with probes complementary to the *HAC1* ORF (B) or spliced junction (C) was performed in duplicate for each strain (wild type is parental isogenic strain BY4741). Expression in (B) was normalized to *ACT1*. Percent spliced *HAC1* in (C) is calculated relative to amount total *HAC1*.
(EPS)

Figure S2 Ypt1 associates with Ada5. (A) *IRE1*-HA strain is viable when grown on YPD+0.5 ug/ml tunicamycin agar plates. Physical interaction in formaldehyde-treated (B,C) or untreated (C) cells between GST-tagged Ypt1 and HA-tagged (B) or TAP-tagged (C) proteins was evaluated by immunopurification with anti-GST conjugated beads and Western blotting. Monoclonal anti-HA (Pierce) or anti-PAP (Sigma Aldrich) antibodies were used for staining. Puf5 served as negative control and Mrs6- as positive control. GST-Ypt1 IP efficiency was verified independently by stripping the blot and re-probing with anti-GST antibody (GE Healthcare) (data not shown). “CH_2_O” = formaldehyde; “−” = untreated cells; “+” = formaldehyde-treated cells.
(EPS)

Figure S3 *ypt1*-DAmP and *sec12*-DAmP block ER export. Accumulation of CPY-precursor (pr) was used as marker for defective ER export. *ypt1*-DAmP (lane 1), *sec12*-DAmP (lane 2), and their isogenic wild type strain (BY4741) (lane 3) were grown at 30°C, while the positive control (*ypt1-3 ts*) and its isogenic wild type (lanes 4–5) were grown at restrictive temperature (37°C) for 3 hrs. Anti-CPY (Invitrogen) was used at 1∶2,000 dilution. Two different exposure times are shown: (A) 2 min and (B) 30 sec. pr = precursor CPY; m = mature CPY.
(EPS)

Figure S4 Ypt1 knockdown has wild type-like Hac1p levels. Quantitative Western blot stained with anti-Hac1 and anti-Gapdh antibodies. *ypt1*-knockdown replicates are in lanes 1–2; wild type replicates are in lanes 3–4. ImageJ was used to quantify the amounts of Hac1p (shown in bar plot next to the Western blot), which were normalized to the expression of the Gapdh protein (loading control).
(EPS)

Figure S5 Unspliced *HAC1* RNA is stabilized in *ada5Δ* and *ire1Δ* strains. *hac1Δ*, *ada5Δ*, and *ire1Δ* strains were transformed with an “unspliceable” *HAC1* variant (see “Materials and Methods”). Cells in (A) and (B) were treated with 3 ug/ml thiolutin for 30 min and quantitative RT-PCR was used to compare expression of *HAC1*u (A) or *ACT1* control (B) in treated versus untreated cells. Values were normalized to GAPDH expression. Data shown are averages of 3 replicates per strain. Asterisks indicate significant differences. * = *p*<0.05 by one-tailed t-test.
(EPS)

Figure S6 Testing UPR induction in *ypt1*-knockdown cells. (A) No obvious growth defect is seen when the *YPT1* mutant is grown on 0.5 ug/ml tunicamycin-containing YPD agar plates. Isogenic parental BY4741 and *ire1Δ* strains are included as controls. (B–E) *ypt1*-DAmP or isogenic BY4741 were treated with 10 mM DTT for indicated times and expression of canonical UPR target genes, *KAR2* (B), *ERO1* (C), *PDI1* (D), or spliced *HAC1* (E), was measured by quantitative RT-PCR. All values are normalized to actin levels and experiments are done in duplicate. (F) Quantitative Western blot using anti-Hac1p polyclonal antibody, showing Hac1p expression 20 min after treatment with 10 mM DTT. Anti-Gapdh antibody (Abcam) is used as loading control. *ypt1*-knockdown replicates are in lanes 1–2; wild type replicates are in lanes 3–4. ImageJ was used to quantify Hac1p amounts and normalize to Gapdh levels (shown below the Western blot in bar plot). Asterisks indicate significant differences. * = *p*<0.05, ** = *p*<0.005 by t-test.
(EPS)

Figure S7 Ypt1 knockdown recovers from UPR slower than wild type. A longer recovery time course (3 hours after wash) was performed to complement data summarized in Figure 4. Data shown are averages of two replicates per strain. Samples were collected at indicated times and expression of spliced *HAC1* was measure by quantitative RT-PCR. All values are normalized to actin levels. Asterisks indicate significant differences. * = *p*<0.05, ** = *p*<0.005 by t-test.
(EPS)

Table S1 Yeast strains used and annotated functions for proteins discussed in the paper.
(XLSX)

Table S2 Summary of protein microarray data from *HAC1* RNA hybridizations. A total of 6 replicate experiments were performed, swapping the Cy dyes used to label *HAC1* RNA and total mRNA for half of the samples. Data are median-centered and z-scores are calculated by subtracting the mean and dividing by the standard deviation. Proteins that met an enrichment threshold (see “Materials and Methods”) are shown in red.
(XLS)

Table S3 Summary of DNA microarray data from GST-Ypt1 RIP-ChIP experiments. IPs of tagged Ypt1 were done under normal conditions (tab 1) or after 50 min treatment with DTT (tab 2). Data are median-centered. Significantly enriched RNAs (by SAM analysis) are shown in tabs 3 and 4.
(XLS)

Table S4 The Ypt1-*HAC1* interaction requires *IRE1*, *ADA5*, and intact *HAC1* 3′UTR. GST-Ypt1 was IPed from *ire1*-(tab 1), *ada5* (tab 2), or *hac1-3′UTRΔ* cells (tab 3). RNAs that are significantly under-enriched in comparison to wild type cells identified by SAM are shown.
(XLS)

Table S5 Ada5 does not interact stably with *HAC1* RNA *in vivo*. HA-Ada5 was IPed from cells and RNAs were identified by DNA microarray analysis (tab 1). Significantly enriched RNAs (relative to a Mock IP with untagged isogenic strain) were identified by SAM (tab 2).
(XLS)

Table S6 Gene expression of *ypt1-* and *sec12*-knockdown strains. UPR targets are annotated by Travers et al.(2000). *Cell* 101. DNA microarrays were used to compare gene expression profiles of knockdown (labeled with Cy5) and isogenic parental wild type (labeled with Cy3) strains. Data are median-centered and z-scores are calculated by subtracting the mean and dividing by the standard deviation.
(XLS)

## References

[1] Cox JS, Walter P (1996). A novel mechanism for regulating activity of a transcription factor that controls the unfolded protein response.. Cell.

[2] Kawahara T, Yanagi H, Yura T, Mori K (1997). Endoplasmic reticulum stress-induced mRNA splicing permits synthesis of transcription factor Hac1p/Ern4p that activates the unfolded protein response.. Molecular biology of the cell.

[3] Travers KJ, Patil CK, Wodicka L, Lockhart DJ, Weissman JS (2000). Functional and genomic analyses reveal an essential coordination between the unfolded protein response and ER-associated degradation.. Cell.

[4] Ruegsegger U, Leber JH, Walter P (2001). Block of HAC1 mRNA translation by long-range base pairing is released by cytoplasmic splicing upon induction of the unfolded protein response.. Cell.

[5] Chapman RE, Walter P (1997). Translational attenuation mediated by an mRNA intron.. Current Biology.

[6] Aragon T, van Anken E, Pincus D, Serafimova IM, Korennykh AV (2009). Messenger RNA targeting to endoplasmic reticulum stress signalling sites.. Nature.

[7] Jedd G, Richardson C, Litt R, Segev N (1995). The Ypt1 GTPase is essential for the first two steps of the yeast secretory pathway.. J Cell Biol.

[8] Chang HJ, Jesch SA, Gaspar ML, Henry SA (2004). Role of the unfolded protein response pathway in secretory stress and regulation of INO1 expression in Saccharomyces cerevisiae.. Genetics.

[9] Higashio H, Kohno K (2002). A genetic link between the unfolded protein response and vesicle formation from the endoplasmic reticulum.. Biochemical and biophysical research communications.

[10] Leber JH, Bernales S, Walter P (2004). IRE1-independent gain control of the unfolded protein response.. PLoS Biol.

[11] Sato M, Sato K, Nakano A (2002). Evidence for the intimate relationship between vesicle budding from the ER and the unfolded protein response.. Biochemical and biophysical research communications.

[12] Tsvetanova NG, Klass DM, Salzman J, Brown PO (2010). Proteome-wide search reveals unexpected RNA-binding proteins in Saccharomyces cerevisiae.. PLoS ONE.

[13] Cherry JM, Ball C, Weng S, Juvik G, Schmidt R (1997). Genetic and physical maps of Saccharomyces cerevisiae.. Nature.

[14] Scherrer T, Femmer C, Schiess R, Aebersold R, Gerber AP (2011). Defining potentially conserved RNA regulons of homologous zinc-finger RNA-binding proteins.. Genome Biol.

[15] Scherrer T, Mittal N, Janga SC, Gerber AP (2010). A screen for RNA-binding proteins in yeast indicates dual functions for many enzymes.. PLoS ONE.

[16] Basyuk E, Galli T, Mougel M, Blanchard JM, Sitbon M (2003). Retroviral genomic RNAs are transported to the plasma membrane by endosomal vesicles.. Developmental cell.

[17] Dollar G, Struckhoff E, Michaud J, Cohen RS (2002). Rab11 polarization of the Drosophila oocyte: a novel link between membrane trafficking, microtubule organization, and oskar mRNA localization and translation.. Development.

[18] Green EG, Ramm E, Riley NM, Spiro DJ, Goldenring JR (1997). Rab11 is associated with transferrin-containing recycling compartments in K562 cells.. Biochem Biophys Res Commun.

[19] Jankovics F, Sinka R, Erdelyi M (2001). An interaction type of genetic screen reveals a role of the Rab11 gene in oskar mRNA localization in the developing Drosophila melanogaster oocyte.. Genetics.

[20] Cao XC, Barlowe C (2000). Asymmetric requirements for a Rab GTPase and SNARE proteins in fusion of COPII vesicles with acceptor membranes.. Journal of Cell Biology.

[21] Richardson CJ, Jones S, Litt RJ, Segev N (1998). GTP hydrolysis is not important for Ypt1 GTPase function in vesicular transport.. Molecular and Cellular Biology.

[22] Calero M, Chen CZ, Zhu W, Winand N, Havas KA (2003). Dual prenylation is required for Rab protein localization and function.. Molecular biology of the cell.

[23] Cao X, Ballew N, Barlowe C (1998). Initial docking of ER-derived vesicles requires Uso1p and Ypt1p but is independent of SNARE proteins.. The EMBO journal.

[24] Stevens T, Esmon B, Schekman R (1982). Early stages in the yeast secretory pathway are required for transport of carboxypeptidase Y to the vacuole.. Cell.

[25] Tenenbaum SA, Carson CC, Lager PJ, Keene JD (2000). Identifying mRNA subsets in messenger ribonucleoprotein complexes by using cDNA arrays.. Proceedings of the National Academy of Sciences of the United States of America.

[26] Tusher VG, Tibshirani R, Chu G (2001). Significance analysis of microarrays applied to the ionizing radiation response.. Proceedings of the National Academy of Sciences of the United States of America.

[27] Grant PA, Schieltz D, Pray-Grant MG, Steger DJ, Reese JC (1998). A subset of TAF(II)s are integral components of the SAGA complex required for nucleosome acetylation and transcriptional stimulation.. Cell.

[28] Welihinda AA, Tirasophon W, Kaufman RJ (2000). The transcriptional co-activator ADA5 is required for HAC1 mRNA processing in vivo.. The Journal of biological chemistry.

[29] Breslow DK, Cameron DM, Collins SR, Schuldiner M, Stewart-Ornstein J (2008). A comprehensive strategy enabling high-resolution functional analysis of the yeast genome.. Nature Methods.

[30] Barlowe C, Schekman R (1993). SEC12 encodes a guanine-nucleotide-exchange factor essential for transport vesicle budding from the ER.. Nature.

[31] d'Enfert C, Wuestehube LJ, Lila T, Schekman R (1991). Sec12p-dependent membrane binding of the small GTP-binding protein Sar1p promotes formation of transport vesicles from the ER.. The Journal of cell biology.

[32] Ogawa N, Mori K (2004). Autoregulation of the HAC1 gene is required for sustained activation of the yeast unfolded protein response.. Genes to cells : devoted to molecular & cellular mechanisms.

[33] Tipper DJ (1973). Inhibition of yeast ribonucleic acid polymerases by thiolutin.. J Bacteriol.

[34] Jimenez A, Tipper DJ, Davies J (1973). Mode of action of thiolutin, an inhibitor of macromolecular synthesis in Saccharomyces cerevisiae.. Antimicrob Agents Chemother.

[35] Wang Y, Liu CL, Storey JD, Tibshirani RJ, Herschlag D (2002). Precision and functional specificity in mRNA decay.. Proc Natl Acad Sci U S A.

[36] Lee KP, Dey M, Neculai D, Cao C, Dever TE (2008). Structure of the dual enzyme Ire1 reveals the basis for catalysis and regulation in nonconventional RNA splicing.. Cell.

[37] Shamu CE, Walter P (1996). Oligomerization and phosphorylation of the Ire1p kinase during intracellular signaling from the endoplasmic reticulum to the nucleus.. The EMBO journal.

[38] Kawahara T, Yanagi H, Yura T, Mori K (1998). Unconventional splicing of HAC1/ERN4 mRNA required for the unfolded protein response. Sequence-specific and non-sequential cleavage of the splice sites.. The Journal of biological chemistry.

[39] Sidrauski C, Cox JS, Walter P (1996). tRNA ligase is required for regulated mRNA splicing in the unfolded protein response.. Cell.

[40] Benli M, Doring F, Robinson DG, Yang X, Gallwitz D (1996). Two GTPase isoforms, Ypt31p and Ypt32p, are essential for Golgi function in yeast.. The EMBO journal.

[41] Gallwitz D, Donath C, Sander C (1983). A yeast gene encoding a protein homologous to the human c-has/bas proto-oncogene product.. Nature.

[42] Matsui Y, Toh-e A (1992). Isolation and characterization of two novel ras superfamily genes in Saccharomyces cerevisiae.. Gene.

[43] Wichmann H, Hengst L, Gallwitz D (1992). Endocytosis in yeast: evidence for the involvement of a small GTP-binding protein (Ypt7p).. Cell.

[44] Beinert H, Kennedy MC (1993). Aconitase, a two-faced protein: enzyme and iron regulatory factor.. Faseb Journal.

[45] Hentze MW (1994). Enzymes as Rna-Binding Proteins - a Role for (Di)Nucleotide-Binding Domains.. Trends in Biochemical Sciences.

[46] Nagy E, Rigby WF (1995). Glyceraldehyde-3-phosphate dehydrogenase selectively binds AU-rich RNA in the NAD(+)-binding region (Rossmann fold).. J Biol Chem.

[47] Zhou Y, Yi X, Stoffer JB, Bonafe N, Gilmore-Hebert M (2008). The multifunctional protein glyceraldehyde-3-phosphate dehydrogenase is both regulated and controls colony-stimulating factor-1 messenger RNA stability in ovarian cancer.. Molecular Cancer Research.

[48] Morsomme P, Riezman H (2002). The Rab GTPase Ypt1p and tethering factors couple protein sorting at the ER to vesicle targeting to the Golgi apparatus.. Developmental cell.

[49] Kamena F, Diefenbacher M, Kilchert C, Schwarz H, Spang A (2008). Ypt1p is essential for retrograde Golgi-ER transport and for Golgi maintenance in S. cerevisiae.. Journal of cell science.

[50] Ullrich O, Reinsch S, Urbe S, Zerial M, Parton RG (1996). Rab11 regulates recycling through the pericentriolar recycling endosome.. The Journal of cell biology.

[51] Gietz D, Woods RA (1998). Transformation of yeast by the lithium acetate single-stranded carrier DNA/PEG method.. Yeast Gene Analysis.

[52] Sikorski RS, Hieter P (1989). A System of Shuttle Vectors and Yeast Host Strains Designed for Efficient Manipulation of DNA in Saccharomyces-Cerevisiae.. Genetics.

[53] Sidrauski C, Walter P (1997). The transmembrane kinase Ire1p is a site-specific endonuclease that initiates mRNA splicing in the unfolded protein response.. Cell.

[54] Knop M, Siegers K, Pereira G, Zachariae W, Winsor B (1999). Epitope tagging of yeast genes using a PCR-based strategy: more tags and improved practical routines.. Yeast.

[55] Hogan DJ, Riordan DP, Gerber AP, Herschlag D, Brown PO (2008). Diverse RNA-binding proteins interact with functionally related sets of RNAs, suggesting an extensive regulatory system.. PLoS Biol.

[56] Oliviero Ca (1993). Current Protocols in Molecular Biology: Preparation of Yeast RNA..

[57] Abramoff MD, Magalhaes PJ, Ram SJ (2004). Image Processing with ImageJ.. Biophotonics International.

